# Screening of latent tuberculosis infection among patients with diabetes mellitus from a high-burden area in Brazil

**DOI:** 10.3389/fcdhc.2022.914574

**Published:** 2022-08-17

**Authors:** Amanda Vital Torres, Raquel da Silva Corrêa, Maria de Fátima Bevilacqua, Luana Cristina França do Prado, Flavia Miranda Gomes de Constantino Bandeira, Luciana Silva Rodrigues, Marilia Brito Gomes

**Affiliations:** ^1^ Diabetes Unit, Department of Internal Medicine, Faculty of Medical Science, Rio de Janeiro State University (UERJ), Rio de Janeiro, RJ, Brazil; ^2^ Laboratory of Immunopathology, Department of Pathology and Laboratories, Faculty of Medical Science, Rio de Janeiro State University (UERJ), Rio de Janeiro, RJ, Brazil; ^3^ Hematology and Transfusion Unit, Department of Internal Medicine, Faculty of Medical Science, Rio de Janeiro State University (UERJ), Rio de Janeiro, RJ, Brazil; ^4^ Herbert de Souza Hemotherapy Service, Pedro Ernesto University Hospital (HUPE), Rio de Janeiro State University (UERJ), Rio de Janeiro, RJ, Brazil

**Keywords:** diabetes, latent tuberculosis infection, QuantiFERON-TB gold in tube, interferon-gamma release assay, diabetes mellitus, tuberculosis

## Abstract

Although several cohort studies have raised the important association between diabetes mellitus (DM) and latent tuberculosis infection (LTBI), evidences are limited and controversial. Furthermore, it is well documented that the poor glycemic control may exacerbate the risk for active TB. Thus, the monitoring of diabetic patients living in high-incidence areas for TB is an important concern in views of available diagnostic tests for LTBI. In this cross-sectional study, we estimate the association of DM and LTBI among diabetic patients classified as type-1 DM (T1D) or type-2 DM (T2D) living in Rio de Janeiro, RJ, Brazil – considered a high TB burden region of these country. Non-DM volunteers were included as endemic area healthy controls. All participants were screened for DM using glycosylated-hemoglobin (HbA_1c_) and for LTBI using the QuantiFERON-TB Gold in Tube (QFT-GIT). Demographic, socioeconomic, clinical and laboratorial data were also assessed. Among 553 included participants, 88 (15.9%) had QFT-GIT positive test, of which 18 (20.5%) were non-DM, 30 (34.1%) T1D and 40 (45.4%) T2D. After adjustments for potential baseline confounders, age, self-reported non-white skin color and an active TB case in the family were significantly associated with LTBI among the studied population by using a hierarchical multivariate logistic regression analysis. Additionally, we verified that T2D patients were able to produce significant increased interferon-gamma (IFN-γ) plasma levels in response to *Mycobacterium tuberculosis*-specific antigens, when compared to non-DM individuals. Altogether, our data showed an increased prevalence of LTBI among DM patients, albeit non-statistically significant, and point out to important independent factors associated with LTBI, which deserve attention in monitoring patients with DM. Moreover, QFT-GIT test seems to be a good tool to screening LTBI in this population, even in a high TB burden area.

## Introduction

Diabetes Mellitus (DM) is a chronic disease with an increasing worldwide prevalence. Nowadays, the disease can be considered an epidemic clinical condition and a huge public health problem because it may lead to a loss in quality of life and early mortality (1, 2). Considering the increased life expectancy reached in the last decades among people with diabetes, a rise in the occurrence of diabetes-related chronic complications is also expected, mainly due to long lasting exposure to hyperglycemia (3). Such complications may include macroangiopathy and microangiopathy (retinopathy, nephropathy and neuropathy) that can compromise an individual’s health and imply in higher expenditures by the public health system. A prompt diagnosis and an adequate treatment can lead to higher chances of obtaining a good glycemic control, which in turn has been proven to reduce diabetes-related complications (4). In addition to these diabetes-related complications, it is currently known that high levels of blood glucose may result in altered immune responses leading to higher susceptibility to several infections, including tuberculosis (TB) (5, 6).

Tuberculosis (TB) is an infectious disease, caused by the *Mycobacterium tuberculosis* (Mtb), which affects primarily the lungs, but can also reach other organs. According to the World Health Organization (WHO), 10 million new cases are diagnosed and 1.2 million of deaths worldwide are registered every year, indicating that TB is a serious public health issue (7). Brazil remains included in the 30 high TB/TB-HIV coinfection burden countries, and in 2019 were registered 66,819 new cases with an incidence rate of 31.6 per 100,000 inhabitants, and 4,500 deaths (mortality rate of 2.2 per 100,000), pointing out Acre, Amazonas and Rio de Janeiro which have shown incidence coefficients above of the national average (8). According WHO, less than half of the cases are notified, which shows a weakness in policies regarding the disease control. This serious condition is due to an increase in poverty, poor distribution of family income and increased urbanization. Many risk factors have been associated to TB, such as male gender, smoking, alcoholism, low body weight, renal diseases, contact with people who have TB, social class and DM. In particular, Brazil has a high prevalence of both diseases (DM and TB), being of paramount importance to investigate reliable and practical diagnostic methods (7).

It is estimated that around one-quarter of the world’s population is asymptomatically infected by Mtb, a condition called *latent tuberculosis infection* (LTBI) and in which individuals are in a high risk of active TB development. Although in an intriguing and poorly understood way, only 5-10% of the people with LTBI progress to the active form of the disease (9), it is a consensus that cell immunity is the main mechanism involved in the protection against Mtb, being interferon-gamma (IFN-γ) production and predominantly type I immune response considered as biomarkers of the protection against TB (10). In views of this last aspect, immunological tests such as tuberculin skin test (TST) and interferon-gamma release assays (IGRA) represent an indirect response to Mtb infection currently used to LTBI diagnosis, even though they are not considered as “gold standard”. Moreover, their performances are limited or compromised due to immunological status and/or populations from high TB burden areas (11).

The present study intended to determine the prevalence of LTBI among type-1 (T1D) and type-2 DM (T2D) patients living in Rio de Janeiro, a Brazilian State that occupies the 2^th^ place in number of TB cases in Brazil showing an incidence rate of 60 per 100,000 (8), by using the commercial IGRA QuantiFERON-TB Gold in Tube (QFT-GIT). Demographic, socioeconomic, clinical and laboratorial data were also assessed to analyze the association with LTBI among study population.

## Methods

### Study design and participants

This was a cross-sectional study conducted with consecutive patients classified as type-1 DM (T1D) and type-2 DM (T2D) patients who were receiving health care from the Brazilian National Health System (SUS), from June 2015 to June 2017 at the Diabetes Unit in the Policlínica Piquet Carneiro, Rio de Janeiro State University (PPC/UERJ), Rio de Janeiro, RJ, Brazil. DM patients were diagnosed according to American Diabetes Association (ADA) criteria (12). All T1D patients have been in continuous use of insulin since their diagnosis, and with at least 6 months of follow-up at this diabetes center. As control subjects, healthy volunteers that were medical staff (administration, nurses or physicians) or blood donors from the Herbert de Souza Hemotherapy Center at Pedro Ernesto University Hospital (HUPE/UERJ) were included in the study and attested/named as *non-DM group*. The exclusion criteria consisted in HIV seropositive patients, pregnant women, lactating women, presence of acute or chronic infectious, patients with diabetic ketoacidosis in the prior three months to the assessment and patients that had difficulties in walking and moving or to go to the hospital for medical care. Blood samples were collected from all participants aiming to measure glycated hemoglobin (HbA_1c_) and to detect LTBI by using QuantiFERON-TB Gold in Tube (QFT-GIT) test. Individuals with HbA_1c_ at 5.7 – 6.5% or indeterminate QFT-GIT were also excluded. The protocols were approved by the Pedro Ernesto University Hospital Ethics Committee (number 686.651) and all participants signed a written informed consent for the study.

### Measures and definitions

Glycemic control in patients with DM were determined by HbA1_c_ levels measured in whole blood collected in ethylenediamine tetra-acetic acid (EDTA) tubes and processed using high-performance liquid chromatographic method (HPLC, Bio-Rad Laboratories, Hercules, California, USA). Adequate glycemic control was defined as the presence of HbA_1c_ levels < 7.0% (58 mmol/mol) (12), and inadequate glycemic control was defined as HbA_1c_ levels ≥ 7.0% (58 mmol/mol). All non-DM participants had HbA_1c_ levels ≤ 5.6%.

The presence of LTBI was determined using the QFT-GIT test (Cellestis Limited, Australia) according to the manufacturer’s instructions. Briefly, 1 mL of whole blood was draw into the three QFT-GIT tubes pre-coated with saline (Nil, negative control), Mtb-specific antigens (ESAT-6, CFP-10 and TB 7.7), or mitogen (positive control) and incubated for 18-24 h at 37°C. After centrifugation, the supernatant was collected and stored frozen at -20°C until IFN-γ measurement, which was done by an enzyme-linked immunosorbent assay (ELISA) from QFT-GIT kit. IFN-γ-Mtb-specific levels were calculated by subtracting of the values obtained from Mtb antigen tubes minus Nil/control tube. QFT-GIT result was defined as positive when IFN-γ in response to Mtb-specific antigens were ≥ 0.35 IU/ml and IFN-γ levels in response to mitogen (mitogen tube minus Nil/control tube) ≥ 0.5 IU/mL. Indeterminate result was defined as IFN-γ of Nil/control > 8.0 IU/mL or positive control value < 0.5 IU/mL. Results were calculated using the manufacturer’s QFT-GIT software.

### Questionnaire

Gender, current age, age at diagnosis, DM time duration, presence of comorbidities, self-reported skin color (according to the Instituto Brasileiro de Geografia e Estatística, IBGE) (13), employment status and years of formal education provided verbally as well as housing conditions and TB exposure history were assessed using medical records or a questionnaire applied by individual interviews during a clinical visit.

Economic status was defined according to the Brazilian Economic Classification Criteria (14), which is based on educational status and house-income and possession of certain house appliances. The following classes of economic status were considered for this analysis: high, middle, low and very low. Regions of housing was defined based on pragmatic areas at Rio de Janeiro State, Brazil.

Body mass index (BMI) was classified as normal, overweight, and obese according to WHO criteria: underweight BMI <18.5 kg/m^2^, normal weight ≥ 18.5 to < 25 kg/m^2^, overweight ≥ 25 to < 30kg/m^2^ and obese as BMI ≥ 30 k/gm^2^ (15). Excess use of alcoholic beverages and current smoking status were defined by self-reported (daily or occasional current user).

### Statistical analysis

A Mann-Whitney or Kruskal-Wallis followed by Dunn’s correction tests were used to compare variables with nonparametric distribution. A T-test or ANOVA (with Sidak correction) was used for parametric distribution. We used Pearson’s univariate correlation when applicable. Categorical variables were reported as percentage and Chi-square tests were used for comparison.

To further explore the association between LTBI (QFT-GIT positivity) and DM status a multivariable hierarchical logistic regression (Backward Wald model) was performed to determine which variables could be associated with the presence of QFT-GIT positivity as dependent variable. To select the independent variables, we chose those with statistical significance in an exploratory analysis or those with clinical plausibility. Afterwards, the order of entry into the model was initially demographic and social data [age, gender, self-reported color-race (stratified as white and non-white), economic classes, years of study] followed by clinical data (DM status, use of statin and antihypertensive drugs and finally data related to TB, such as familiar case of TB). DM status entered in the model first as yes/no and secondly stratified as controls (non-DM), T1D and T2D patients. The model fit was assessed through a Hosmer and Lemeshow and Omnibus test. The calculated Nagelkerke R^2^ and the odds ratio (OR) with a 95% confidence interval (CI) were expressed as indicated. All statistical analyzes were performed with a 95% confidence interval (CI) and the significance level was P ≤ 0.05. Statistical Package for Social Sciences (SPSS) version 16.0. GraphPad Prism version 9 was used for graphic illustrations.

## Results

### Overview of the study population

**Figure 1:**
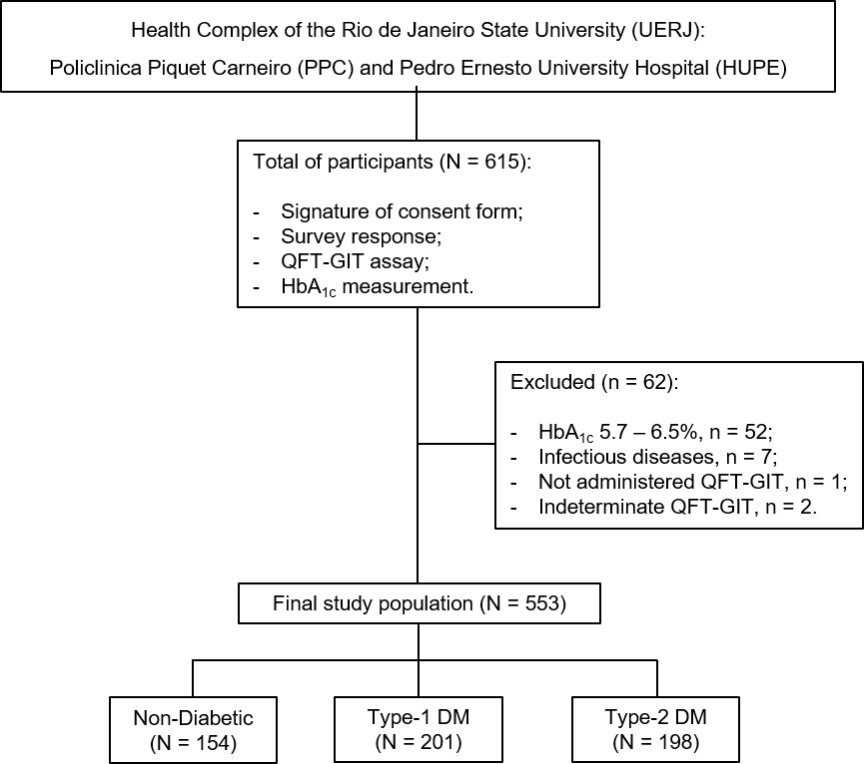
Flow chart of the study design. A total of 615 individuals were recruited for the study. Of these, 553 represented the final study population based on the eligible criteria and were categorized as follow, non-DM subjects (N = 154), type-1 DM (N = 201) and type-2 DM (N = 198). DM, diabetes mellitus; HbA_1c_, glycosylated hemoglobin; QFT-GIT, QuantiFERON-TB Gold in Tube; n, sample number.

**Figure 1** shows the flow chart of the study design. A total of 615 volunteers was recruited at the Health Complex of Rio de Janeiro State University (UERJ), Rio de Janeiro, RJ, Brazil. We excluded participants presenting HIV, syphilis and hepatitis B or C (n = 7), those who had an indeterminate QFT-GIT (n = 2) or who did not take this test (n = 1). Also, all non-DM subjects showing HbA_1c_ levels among 5.7 – 6.5% were excluded (n = 52). Therefore, a final study population consisted of 553 participants, categorized as follow: i) non-DM (n = 154); ii) T1D (n = 201) and iii) T2D (n = 198).

Baseline characteristics including demographics, socioeconomic, clinical and laboratorial features of the study population are shown in **Table 1**. T1D patients had higher levels of fasting glycemia (197.89 ± 105.58 mg/dL) and HbA_1c_ levels (9.0 ± 2.1%) than patients with T2D (154.29 ± 66.08 mg/dL; 8.33 ± 1.93%, respectively). Patients with T2D were more likely to be older (58.5 ± 9.79 years) and have overweight (35.9%) or obesity (47%) compared to those T1D or non-DM participants. Also, among T2D, 66.7% reported as non-white, 16.7% as current users of cigarettes and 30.8% of the patients were retired. Most of the participants reported not being a current smoker (89.3%) or alcohol user (70.5%).

**Table 1:** Baseline characteristics of study population.

Variable	Non-DM (n = 154)	Type-1 DM (n = 201)	Type-2 DM (n = 198)	*P value*
***Demographic characteristics***				
Age, y	34.6 ± 12.9	32.5 ± 15.0	57.70 ± 10.7	< 0.001
Gender, (%)				
Male	86 (55.8)	97 (50.0)	103 (50.2)	0.580
Self-reported skin color, n (%)				
White	95 (61.7)	79 (40.7)	67 (32.7)	< 0.001
Non-white	59 (38.3)	115 (59.3)	138 (67.3)	
***Socioeconomic characteristics***				
Years of study^a^, n (%)				
0 < 12	18 (11.7)	75 (38.7)	133 (64.9)	< 0.001
> 12	136 (88.3)	119 (61.3)	72 (35.1)	
Occupation, n (%)				
Unemployed	7 (4.4)	26 (13.4)	51 (24.9)	< 0.001
Employed	120(75)	99 (51.0)	90(43.9)	
Student	32 (20.0)	60 (30.9)	3 (1.5)	
Retired	1 (0.6)	9 (4.5)	61 (29.8)	
Economic class^b^, n (%)				
Very low	12 (7.8)	22 (11.3)	41 (20.0)	< 0.001
Low	107 (69.5)	135 (69.6)	143 (69.8)	
Middle	35 (22.7)	37 (19.1)	21 (10.2)	
Regions/Housing, n (%)				0.142
RJ city/Metropolitan	152 (98.7)	199 (99.0)	204 (99.5)	
**Clinical characteristics**				
Duration of diabetes, y	NA	14.5 ± 10.3	12.0 ± 7. 7	< 0.001
Fasting glycemia (mg/dL)				
Mean ± SD	NA	197.89 ± 105.58	154.29 ± 66.08	< 0.001
HbA_1c_, (%)				
Mean ± SD	5.18 ± 0.34	9.1 ± 2.10	8.3 ± 1.9	< 0.001
< 7.0	NA	37 (18.4)	50 (25.30)	< 0.001
HbA_1c_, (mmmol/mol)	33.3 ± 2.1	76.0 ± 10.2	67.2 ± 10.0	< 0.001
BMI (kg/m^2^)	26.2 ± 4.3	23.9 ± 4.3	26.7 ± 4.3	
Underweight (< 18.5)	0	21 (10.8)	2 (1.0)	< 0.001
Normal weight (18.5 – 24.9)	68 (44.2)	97 (50.0)	33 (16.1)	
Overweight (25 – 29.9)	62 (40.3)	59 (30.4)	74 (36.1)	
Obesity (≥ 30.0)	24 (15.6)	17 (8.8)	96 (46.8)	
Current smoker, yes, n (%)	12 (7.8)	15 (7.7)	33 (16.1)	0.001
Alcohol use, yes, n (%)	51 (33.1)	35 (18.0)	46 (22.4)	< 0.001
Medicine/drugs, yes, n (%)				
Hypoglycemicagents	0	43 (22.2)	165 (80.5)	< 0.001
Antihypertensives	12 (7.8)	57 (29.4)	146 (71.2)	< 0.001
Insulin	0	194 (100.0)	82 (40.0)	< 0.001
Corticoid	1 (0.6)	7 (3.6)	11 (5.4)	0.052
Statin	1 (0.6)	42 (21.6)	141 (69.8)	< 0.001
BCG vaccine, yes, n (%)	142 (92.2)	186 (95.9)	184 (89.8)	0.01
Family TB Case, yes, n (%)	26 (16.9)	43 (22.2)	69 (33.8)	0.001
TB Household contact, yes, n (%)	9 (5.8)	15 (7.7)	23 (11.2)	0.2

y, year; data are presented as number (percentage), mean ± SD (standard deviation); DM, Diabetes Mellitus; RJ, Rio de Janeiro, Brazil; HbA_1c_, glycosylated hemoglobin; BCG, Bacillus Calmette-Guérin vaccine; NA, not applied; TB, tuberculosis; BMI, body mass index; n, sample number. P value ≤ 0.05 were considered significant.

a Years of study, 0-5 years, Illiterate and/or Incomplete Elementary School; 6-12 years, Complete Elementar School and/or Complete High School; > 12 years, Complete/Incomplete Higher Education and/or Post-graduation.

b Economic class, Very low, social class D and E; Low, social class C1 and C2; Middle-High, A, B1 and B2 classes.

Regarding the origin of residence and some housing conditions, we verified that the majority of the study population were from very low or low socioeconomic classes’ mainly patients with T2D (89.8%). The most common areas of origin were from Rio de Janeiro districts/Metropolitan area (99.3%). Around one third of all DM patients reported to have a TB case in the family. However, individuals who were in household contact with TB case were not statistically significant among the groups (**Table 1**).

### Prevalence of latent tuberculosis infection among study population

**Figure 2:**
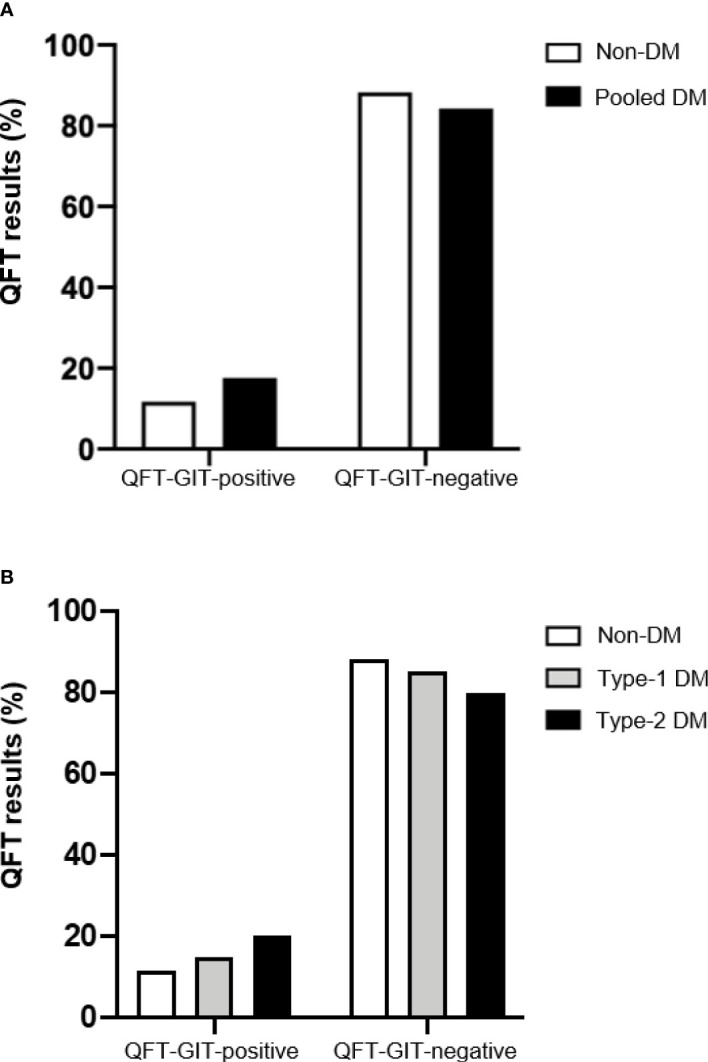
QFT-GIT results between non-diabetic and diabetic patients. The QuantiFERON-TB Gold in Tube results in subjects with Diabetes Mellitus or not were provided by the stimulation of whole blood with Mtb-specific antigens (ESAT-6, CFP-10 and TB7.7) or mitogen (positive control). **(A)** QFT-GIT results (%) from non-DM and pooled MD (T1D and T2D patients) were compared. **(B)** QFT-GIT results (%) from non-DM, T1D and T2D were compared. DM, Diabetes Mellitus; T1D, type-1 DM; T2D, type-2 DM.

Based on the QFT-GIT positivity, the overall prevalence of LTBI among study population was 15.9% (n = 88), of which 18 (20.5%) were non-DM, 30 (34.1%) T1D and 40 (45.5%) T2D (**Table 2**). However, we did not observe a significant association of QFT-GIT positivity with DM status. As shown in **Figure 2**, an increasing prevalence of LTBI, albeit non-statistically significant, was observed in all DM (17.5% [n = 70]) or when DM group was stratified at T1D (15%) and T2D (20.2%) in comparison to non-DM group (11.7%) (**Figures 2A, B**).

**Table 2:** Characteristics of participants with LTBI among study population.

Variable	QFT-positive (n = 88; 15.9%)	QFT-negative (n = 465; 84.1%)	*P value*
***Demographic characteristics***			
Age, y	47.8 ± 17.0	41.40 ± 17.4	<0.001
Gender, male, n (%)	46 (52.3)	225 (48.4)	0.9
Self-reported skin color, n (%)			0.001
White	24 (27.3)	217 (46.7)	
Non-white	64 (72.7)	248 (53.3)	
***Socioeconomic characteristics***			
Years of study^a^, n (%)			0.1
0 - 12	41 (46.6)	185 (39.8)	
> 12	47 (53.4)	280 (60.2)	
Occupation, n (%)			0.09
Unemployed	13 (14.8)	71 (15.3)	
Student	8 (9.1)	87 (18.7)	
Employed	51 (58.0)	252 (54.2)	
Economic class^b^, n (%)			0.115
Very low	17 (22.4)	59 (77.6)	
Low	62 (15.9)	327 (84.1)	
Middle-high	10 (10.6)	84 (89.4)	
Regions/Housing, n (%)			0.9
RJ city and metropolitan regions	87 (98.9)	461 (99.1)	
**Clinical characteristics**			
Clinical status, yes, n (%)			0.1
Non-DM	18 (20.5)	136 (29.2)	
Type-1 DM	30 (34.1)	164 (35.3)	
Type-2 DM	40 (45.4)	165 (35.5)	
BMI (kg/m^2^)	26.8 ± 5.2	26.6± 5.5	0.8
Fasting glycemia (mg/dL)	156.82 ± 92.01	151.63 ± 105.81	0.7
HbA_1c_ ≥ 7,0, (%)	7.8 ± 1.9	7.6 ± 2.40	0.6
HbA_1c_ (mmol)	26.82 ± 5.17	26.66 ± 5.43	0.6
Smoking, Yes (%)	6 (6.8)	29 (6.3)	0.9
Alcoholism, yes, n (%)	18 (13.8)	114 (24.5)	0.3
Drugs, yes, n (%)			
Hypoglycemicagents	34 (38.6)	174 (36.6)	0.9
Antihypertensives	45 (51.1)	170 (36.6)	0.01
Insulin	52 (59.1)	265 (57.0)	0.7
Corticoid	3 (3.4)	16 (3.4)	0.9
Statin	37 (42.7)	147 (31.6)	0.06
BCG vaccine, yes, n (%)	82 (93.2)	430 (92.5)	0.8
Family TB Case, yes, n (%)	31 (35.2)	107 (23.0)	0.04
TB Household contact, yes, n (%)	11 (12.5)	36 (7.7)	0.3

y, year; data are presented as number (percentage), mean ± SD (standard deviation); DM, Diabetes Mellitus; RJ: Rio de Janeiro, Brazil, HbA_1c_, glycosylated hemoglobin, BCG: Bacillus Calmette-Guérin vaccine, TB: tuberculosis, BMI: body mass index, n: sample number. P value ≤ 0.05 were considered significant.

a Years of study: 0-5 years: Illiterate and/or Incomplete Elementary School; 6-12 years: Complete Elementar School and/or Complete High School; > 12 years: Complete/Incomplete Higher Education and/or Post-graduation.

b Economic class: Very low: social class D and E; Low: social class C1 and C2; Middle-High: A, B1 and B2 classes.

P value ≤ 0.05 were considered significant.

As depicted in **Table 2**, among all analyzed variables, LTBI was significantly associated with individuals more likely to be older (mean age = 47.8 ± 17.0 years; p < 0.001), non-white self-report skin color (72.7%; p = 0.001), use of antihypertensive drugs (51.1%; p = 0.01) and the presence of TB case (35.2%; p = 0.04). In the hierarchical multivariable logistic model examining all selected independent variables, we observed that there was a significant association between LTBI prevalence and age (adjusted odds ratio [aOR] 1.018, 95% confidence interval [CI] 1.004 – 1.033; p = 0.01), self-reported skin color non-white (aOR 2.38, 95% CI 1.427 – 3.999; p = 0.001) and a TB case in family (aOR 1.638, 95% CI 0.999 – 2.710; p = 0.05). No association was noted concerning the other variables, including status of DM (**Table 3**). All the independent variables which entered in the model could explain 7.6% (Nagelkerke R-squared) of a given patient having QFT-GIT positivity.

**Table 3:** Characteristics associated with QFT-GIT positivity among study population.

Variable	aOR (95% CI)	*P value*
Age	1.018 (1.004 – 1.033)	0.01
Self-reported skin color, non-white	2.389 (1.427 – 3.999)	0.001
Family TB case, Yes	1.638 (0.999- 2.710)	0.05

QFT-GIT, QuantiFERON TB Gold in Tube; aOR, adjusted Odds Ratio; CI, confidence interval.

P value ≤ 0.05 were considered significant.

Additionally, based on the survey response, we observed that among non-DM participants who were QFT-GIT-positives, 22.2% (4/18) reported a TB case in the family. Among T1D patients QFT-GIT-positive, 40% (12/30) reported a TB case in the family and 13.3% (4/30) had a household contact with a TB index case. Finally, in the T2D group who were positive for QFT-GIT, 40.0% (16/40) individuals reported a TB case in the family and 17.5% (7/40) of them had a household contact with a TB index case (data not shown).

### Interferon-gamma-Mtb-specific plasma levels among diabetic patients

**Figure 3:**
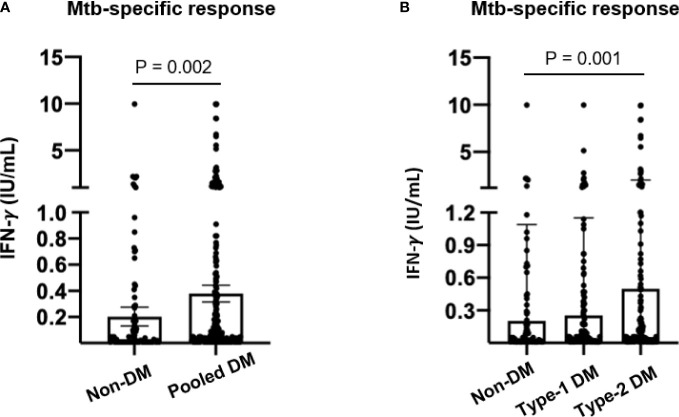
Interferon-gamma plasma levels in response to Mtb-specific antigens by non-diabetic and diabetic patients. Whole blood from study population was stimulated or not with Mtb-specific antigens (ESAT-6, CFP-10 and TB7.7) by using QuantiFERON-TB Gold in Tube (QFT-GIT) assay for 18- 24h at 37°C. A) IFN-g-Mtb-specific levels were compared among study population: **(A)** non-DM and pooled MD (T1D and T2D patients) or **(B)** non-DM, T1D and T2D. IFN-γ. Mtb-specific levels were determined by subtraction between Mtb-specific antigens minus control tube (Nil). Each dot represents an individual value. DM, Diabetes Mellitus; T1D, type-1 DM; T2D, type-2 DM.

As a last exploratory analysis, we investigated the IFN-γ levels produced in response to Mtb specific-antigens in plasma supernatants from QFT-GIT. Significant increased IFN-γ levels were observed in DM patients in comparison to non-DM subjects (0.37 IU/mL *versus* 0.20 IU/mL, respectively; p = 0.0026) (**Figure 3A**). However, when DM group was stratified, only T2D showed significant IFN-γ-Mtb-specific levels in comparison to non-DM (0.50 IU/mL; p = 0.0019) (**Figure 3B**).

In conclusion, our data have shown an increased prevalence of LTBI among DM patients by using IGRA test QFT-GIT, and it points to a significant association with sociodemographic and status of exposition to TB cases as important risk factors for LTBI. Moreover, a quantitative analysis from QFT-GIT revealed that T2D patients produced significant high levels of IFN-γ in response to Mtb-specific antigens.

## Discussion

Despite of DM is a well-recognized risk factor for active tuberculosis, there are heterogeneous and limited evidences in the literature for their association with latent TB infection, LTBI (16, 17). To our best knowledge, the present study is the first one to compare the prevalence of LTBI in individuals with and without DM from a high endemic area in Brazil, the Rio de Janeiro State. Our data showed that albeit non-significant, the prevalence of LTBI among T1D and T2D individuals was increased in comparison to non-DM individual. QFT-GIT positivity was associated with sociodemographic determinants such as age and self-reported skin color (non-white). As expected, having an active TB case in the family was also an important significant variable associated with LTBI. Interestingly, IFN-γ Mtb-specific levels were significantly increased in patients with T2D. Altogether, these results revealed important variables to take into account in the management of DM patients in the screening of LTBI.

DM and TB can affect each other by many mechanisms including monocyte traffic modulation, phagocytosis, cytokine production as well as altered functions of the innate and adaptative immune cells (18). DM increases the risk of TB as well as its severity in case of active disease and has a negative impact on public health, especially in countries where both conditions are strongly predominant (19). The hyperglycemic state is strongly related to the alteration in the expression of receptors of activation and recognition, also for the phagocytic and microbicidal activity of cells of the innate immune system, as well as in the production of cytokines/chemokines and mechanisms of activation of cells of adaptive immunity, mainly in cellular response that are determinant for the response to Mtb (20).

There are no gold standard tests for the diagnosis of LTBI, and those available consist of indirect measures of the cellular immune response to Mtb antigens as the tuberculin skin test (TST) and the IGRAs (T-SPOT and QFT-GIT). It is well documented that prior BCG vaccination, nontuberculous mycobacterium (NMT) or others mycobacteria such as *M. leprae* (etiological agent of leprosy) infection are important causes of false-positive TST results (11). However, TST is widely used in Brazil and other countries due to be considered an inexpensive and accessible method for LTBI detection in adults and active disease in children (21). In the other hand, IGRAs offers numerous advantages over TST, such as reducing professional interference, measuring the specific response to Mtb antigens that are not shared with BCG or most species of NMT (22), in addition to requiring only one visit to the laboratory to be performed. However, it consists of a high-cost test and needs a laboratory structure to be performed (11). In this study, we decided to evaluate the QFT-GIT, the only commercial IGRA available in Brazil, based on their advantages over the TST. All individuals in the present study showed higher levels of IFN-γ in response to mitogen stimuli, which guarantee the validation of the QFT-GIT (data not shown).

Patients with DM recruited for this study were from the Diabetes Outpatient Clinic of the PPC/UERJ, which receives patients from all over the State of Rio de Janeiro, but mostly residents in Rio de Janeiro city, followed by metropolitan regions including Baixada Fluminense and other regions. A predominance of active TB in young adult men (24-35 years) is well established (7, 23). However, studies aiming to establish the predominance of this age range regarding LTBI are scarce and the majority of these studies have been performed in individuals with risk factors such as HIV positivity, health care professionals, people that have close contact with infected patients, use of TNF blockers (24–29). Although some of these studies have identified male gender risk factor for LTBI (29, 30), in our study gender was not related to LTBI **(** **Table 2** **)**. Further studies are necessary to establish the relationship between LTBI and gender in Brazilian population. Interestingly, Brazilian cross-sectional studies have demonstrated an age transition of TB disease incidence as well as LTBI prevalence among healthy individuals to over forty-fifty years (30, 31). Our results are consistent with these particular studies since that QFT-GIT positive individuals identified in the present study presented a mean age of 47.8 (**Table 2**).

The increased risk of LTBI infection among patients with DM in TB-endemic areas is recognized, but yet displaying limited and controversial evidences regarding several aspects, such as the choice or available LTBI diagnostic test, analytical methods employed, study population and TB setting (32). Some evidences from literature support this affirmative, as well as our data which have shown increased proportion of LTBI among DM patients, although not statistically significant. An epidemiological study carried out in Malaysia, a Southeast Asian country with a high incidence of TB (92/100,000 population) observed a similar prevalence of LTBI among diabetic (28.5%) and non-diabetic subjects (29.2%) (33). In addition, even in a high endemic setting, no significant differences were observed in the proportion of LTBI between TB household contacts and individuals without previous TB exposure. A systematic review and metanalysis including 13 observational studies, which were conducted in high-risk populations, such as household contacts of active pulmonary TB, immigrants and immunocompromised patients, revealed a small statistically significant association between DM and risk for LTBI (34). It is worth to note that the studies included in this metanalysis not necessarily show a specific cohort composed by DM patients, as addressed in the present one. Moreover, DM diagnosis was self-reported in the majority of the studies and LTBI investigation was based on different immunological methods, such as TST, QFT-GIT and T.SPOT. Also, status or type of DM is not mentioned. Thus, we emphasize that there are few studies addressing this subject in regions with high incidence of TB. The present study suggests that in Brazil, and particularly in the Rio de Janeiro where there is a high incidence of TB (8), the presence of DM may not represent an isolated risk factor for LTBI and other variables, probably related to social determinants such as those described below, should be investigated.

There is substantial evidence that social variables such as being illiterate, unemployed and belonging to low income stratum are strongly associated with TB (35). TB, poverty and poor access to health care services are strongly linked, which could be of great concern when TB is associated with DM. Nevertheless, a previous study has shown that the prevalence of the TB-DM association was quite similar in both developing and developed countries (36, 37). We have shown that the majority of patients with DM in this study presented 6–12 years of study, considered a medium degree of school attendance, which showed a significant association with LTBI.

Our findings showed a strong association of LTBI among individuals who reported the presence of TB cases in the family. This highlights the need of effective and low cost LTBI screening in those who were known to be exposed to a major risk factor for early LTBI and also active cases detection in this population (38). As we know, patients in contact with TB are included in the high risk group for developing the active form of the disease (17). TB is acquired not only where people live but also where they work or socialize and poverty seems to influence the risk of progression to the disease as well as the risk of infection (37). Socioeconomic characteristics and less favorable neighborhoods where patients reside are independent risk factors for TB (38). Although, the prevalence of TB in metropolitan areas in RJ is higher, we did not observe significant differences regarding the QFT-GIT positivity among different regions of RJ.

In agreement with our data, which points out an increased prevalence of LTBI among T2D patients (**Table 2** and **Figure 2**), some recent studies also have been reported the association of T2D patients and risk factors of LTBI (39, 40). Also, we have observed that T2D patients have produced significant high levels of IFN-γ in response to Mtb-specific antigens from QFT-GIT when compared to non-DM individuals (**Figure 3B**). Active TB patients with DM display higher levels of circulating type-1 (Th1) cytokines such as IFN-γ and tumor necrosis factor (TNF) in comparison to TB patients without DM (41), while LTBI in the presence of DM or pre-DM were associated with reduced levels of these important mediators which are involved in the control of Mtb (42). A recent study conducted in Brazil have shown altered surface molecules (HLA-DR, CD80 and CD86), cytokine/chemokine production and diminished bacterial clearance in monocyte-derived macrophages from T2D infected with Mtb clinical isolates in comparison to healthy individuals (43), suggesting that this group of DM patient may failure in the control of Mtb infection. Alternatively, the Mtb infection may influence the adipose tissue toward to pro-inflammatory cytokines production which in turn impact host metabolic homeostasis (44), adding new views of TB-DM interaction.

Particular strengths of our study are the population of diabetes cases in a large sample of Brazilian patients with T1D and T2D from a wide range of ethnic groups according to self-reported color-race, diagnosed in a community setting. All participating individuals followed a uniform and standardized protocol and similar to other population studies, we used a clinical definition of T1D and T2D assigned by healthcare providers and supported by the quantification of fasting plasma glucose and HbA_1c_. There are some limitations in our study that must be mentioned such as the low availability of TST test during the recruitment period of the study, which is widely distributed by the Health Ministry in Brazil. This fact did not allow us to perform additional comparisons with QFT-GIT in the studied population. Moreover, we could not associate the QFT-GIT findings with clinical evaluation and chest x-ray performance in order to obtain a greater follow-up of the studied population.

In conclusion, although we did not find a statistically significant association between DM and LTBI, this study revealed increased proportion of LTBI prevalence among DM patients from a high-endemic area in Brazil. Sociodemographic variables such as age, self-reported non-white skin color and the presence of TB index case in the family were highlighted as independent risk factors for LTBI. Overall, our data provide important evidences for guide LTBI screening programs among diabetic patients in high-burden TB setting.

## Data availability statement

The raw data supporting the conclusions of this article will be made available by the authors, without undue reservation.

## Ethics statement

The studies involving human participants were reviewed and approved by Hospital Universitário Pedro Ernesto, Universidade do Estado do Rio de Janeiro. The patients/participants provided their written informed consent to participate in this study.

## Author contributions

AT, RS, MB and LF contributed with sample collection and processing, experiments, rationale for the study and manuscript preparation. LR and MB designed the study. Project supervision was performed by LR and MB. LR and MB were responsible for funding acquisition. AT, FG, RS, LF were responsible for participants’ recruitment. AT, RS, LR and MB contributed for data analysis and graph generation. AT, LR and MB analyzed the results. LR and MB were responsible for manuscript revision. AT, RS, LR and MB were responsible for writing the original manuscript draft of the manuscript while MB, LF and FG were in charge of revising it. All authors contributed to the article and approved the submitted version.

## Funding

This work was supported by grants awarded by the Fundação Carlos Chagas Filho de Amparo Pesquisa do Estado do Rio de Janeiro to MBG (FAPERJ; E-26/010.001534/2014). AT was a recipient of a fellowship from CNPq.

## Acknowledgments

The authors are grateful to physicians and nursing staff from Diabetes Unit, PPC/HUPE/UERJ and also to Dr. Walter Costa and Dr. Ana Paula Gomes from TB outpatient at HUPE/UERJ, and Carlos Antonio Negrato for editing the text.

## Conflict of interest

The authors declare that the research was conducted in the absence of any commercial or financial relationships that could be construed as a potential conflict of interest.

## Publisher’s note

All claims expressed in this article are solely those of the authors and do not necessarily represent those of their affiliated organizations, or those of the publisher, the editors and the reviewers. Any product that may be evaluated in this article, or claim that may be made by its manufacturer, is not guaranteed or endorsed by the publisher.
